# Brief action planning targeting prognostic factors for an adult with persistent low back pain without radiculopathy: A case report

**DOI:** 10.1002/ccr3.3280

**Published:** 2020-08-30

**Authors:** Gaelan Connell, Leslie Verville, Carol Cancelliere, Jessica J. Wong, Hainan Yu, Heather M. Shearer

**Affiliations:** ^1^ Faculty of Health Sciences Ontario Tech University Oshawa ON Canada; ^2^ Centre for Disability Prevention and Rehabilitation Canadian Memorial Chiropractic College Ontario Tech University Toronto ON Canada; ^3^ Rehabilitation Sciences Faculty of Medicine University of British Columbia Vancouver BC Canada; ^4^ Epidemiology Division Dalla Lana School of Public Health University of Toronto Toronto ON Canada; ^5^ Institute of Health Policy, Management and Evaluation University of Toronto Toronto ON Canada

**Keywords:** behavioral medicine, heath maintenance, patient education, social care

## Abstract

We describe the implementation of brief action planning in conjunction with evidence‐based clinical practice guideline recommendations to improve self‐efficacy in a patient with psychosocial barriers and persistent nonspecific low back pain.

## Graphical abstract

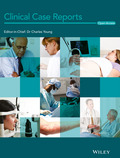

## 1 INTRODUCTION

More than 80% of the Canadian population will experience low back pain (LBP) throughout their lifetime.^1^ While most cases of LBP are benign and self‐limiting, persistent LBP occurs in roughly 20% of the Canadian population.^2^ There is an increased likelihood of delayed recovery in patients with poor prognostic factors such as passive coping strategies, open litigation or compensation claims, unemployment, depression, and high disability levels.^3^, ^4^ Key guideline recommendations for the management of persistent nonspecific LBP include reassurance, education, and emphasizing active versus passive interventions.^3^ Active interventions include recommendations of self‐management strategies for patients with persistent LBP, particularly to foster healthy lifestyle changes in those with related comorbidities.^5^ Collaboration between healthcare providers and patients in the form of shared decision‐making and goal setting can help reinforce the importance of self‐management strategies.

A systematic review of clinical practice guidelines for the management of persistent nonspecific LBP recommends that clinicians identify and address psychosocial barriers to recovery throughout the course of management.^3^ Psychologically informed practice is an approach that uses the routine and specific consideration of psychosocial barriers and aims to prevent the development of pain‐related activity limitations.^6^ Psychological interventions such as education, goal setting, coping techniques, and behavioral interventions for patients experiencing LBP have been associated with moderately improved pain intensity compared with waitlist controls.^7^ Brief action planning is a behavioral intervention that is influenced by principles of motivational interviewing and behavior change theory.^8^ Motivational interviewing and behavior change theory can be used to promote active participation and engagement in treatment recommendations. Brief action planning is a self‐management technique that follows a structured series of questions to facilitate goal setting and action planning between the patient and provider.^9^ In this paper, we present the implementation of a brief action plan in conjunction with evidence‐based clinical practice guideline recommendations for a patient experiencing persistent nonspecific LBP and psychosocial barriers.

## 2 CASE PRESENTATION

We obtained written informed consent from the patient to publish this case report.

### 2.1 Case history and examination

An unemployed 28‐year‐old female patient presented to her healthcare provider with a primary complaint of persistent nonspecific low back pain without radiculopathy and secondary complaints of neck and upper back discomfort. Persistent nonspecific low back pain is defined as symptoms lasting >3 months and is not caused by a recognizable specific pathology such as fracture, infection, malignancy, or inflammatory spondyloarthropathy.^10^ She reported that the onset of her symptoms began over twelve years ago and was relieved with heat and rest. She was injured in three rear‐end collisions over the past two years. Prior to the motor vehicle accidents, she reported psychological stressors, including diagnosed clinical depression and post‐traumatic stress disorder. She also noted symptoms of obsessive‐compulsive disorder and disordered eating. This patient was receiving ongoing treatment from a dietician and a psychiatrist. She described that leaving her home and performing daily activities were difficult due to apprehension of LBP exacerbation. Upon clinical examination, there were no notable findings on observation or gait. Her lumbar range of motion was full but painful in extension. Her lower limb neurological assessment was normal. Additionally, her sacroiliac joint provocation testing was painful bilaterally. She reported a 7/10 on the numeric pain rating scale before participating in brief action planning.

History‐taking identified poor prognostic factors such as increased psychological stressors suspected to delay recovery. Therefore, several educational strategies were used during the course of treatment to address this. The initial patient interaction began with patient‐centered communication. This included giving the patient time to tell her story and providing a nonjudgmental environment, reassuring that hurt does not equal harm and that her body was resilient, promoting movement and work, and avoiding bed rest.^11^ Altogether, this information led to identifying and implementing patient‐centered goals around home‐based exercise as part of brief action planning.

### 2.2 Brief action planning

Based on her previous episodes of back pain, she described having temporary relief of symptoms with manual therapy, including spinal manipulation but continued to experience recurrent pain. Brief action planning can be used as part of a multifactorial treatment strategy and has been used to build self‐efficacy in the management of chronic illness and exercise adherence.^9^, ^12^ The process of action planning can improve self‐efficacy, which is defined as the confidence in one's ability to enact a behavior.^9^ Brief action planning is a useful behavior change technique during clinical encounters as it provides a structured and concise approach.^8^ Agenda setting, establishing SMART (Specific, Measurable, Achievable, Realistic, and Timely) goals, scaling for confidence, and follow‐up are crucial components of brief action planning. In circumstances where patients demonstrate low confidence or require more support, using a patient‐approved behavioral menu enables practitioners to share ideas with the patient.^9^ A behavioral menu is a strategy whereby the clinician suggests two or three ideas to help the patient discover their own goals.

According to recent clinical practice guidelines, manual therapy, exercise, and patient education are recommended for the management of persistent nonspecific LBP. Based on the evidence, it was appropriate to incorporate these into the current patient's plan of management.^3^, ^7^, ^13^ This patient underwent four treatment sessions with the chiropractor over two months each including spinal manipulative therapy, soft‐tissue therapy, education, reassurance, and brief action planning. During the first visit, the patient stated her goal of feeling stronger and more resilient. She described that squats were an activity that felt comfortable and that she had experienced success in the past. At the end of the visit, she was asked, “Is there anything you'd like to do for your health in the next week or two?”. She set a personal goal of performing 100 squats per day, four days per week in her apartment gym. She felt confident that she could accomplish this goal and began the following day. Other self‐management strategies were reviewed during this session, including breathing techniques and stretches. During her follow‐up appointment two weeks later, she reported adherence to her plan. The clinician acknowledged her success, and the patient reported that she would like to sustain this goal over a longer period of time. She reported feeling low back pain a few days prior to her appointment but felt that she was able to manage symptoms on her own, using heat, stretches, and breathing techniques.

During a follow‐up appointment for a subsequent episode of low back pain, that occurred nearly two months after the introduction of the initial brief action plan, we built on her previous goal and recorded a more detailed account of our interaction (described below).

Four steps for conducting a brief action planning are as follows:

Step 1. Agenda Setting

This interaction begins by eliciting a behavioral focus or goal from the patient. This step encourages patients to clarify which factors are relevant to them and why.^14^ If the patient is unsure, this is an opportunity to offer a menu of options or a behavioral menu which allows patients to select a behavior to address.^9^, ^15^

Practitioner: Is there anything you would like to do to improve your low back pain in the next week or two?

Patient: I would like to have 30 minutes of physical activity per day.

Step 2. SMART Planning

Important questions relating to the plan include “what?” “where?” “when?” “how long?” “how often?” “how much?” and “when will you start?”.^9^ Ideally, the target should be achievable by the next consultation.^14^ Ask the patient to elicit a commitment statement by repeating the specifics of the plan as this may lead to unconscious self‐reflection about the feasibility of the plan.^9^

Practitioner: Great! Where will you be exercising?

Patient: In my apartment gym.

Practitioner: What specific exercises do you plan to do? Will you be doing cardio or strength exercises?

Patient: I will exercise on the treadmill or elliptical, followed by low back rehab exercises like squats and clamshells.

Practitioner: When will you do these exercises?

Patient: Every day in the morning.

Practitioner: When would you like to start this plan?

Patient: Tomorrow.

Practitioner: Can you repeat the plan to me, so that I know we are on the same page?

Patient: I will be doing the elliptical or treadmill and rehab exercises in my apartment gym for 30 minutes everyday starting tomorrow

Step 3. Scaling for Confidence

Ask the patient how confident they are in carrying out their plan on a scale of 0‐10 after a commitment statement has been elicited.^9^ Zero is defined as not confident at all, and ten is defined as completely confident.^9^ Scaling questions assess the importance and confidence of changing the target behavior. Assessing readiness to change also provides an opportunity for the patient to demonstrate uncertainty, ambivalence, or a lack of readiness.^14^ Low confidence can be characterized as below 7/10 and impacts self‐efficacy.^9^ When confidence is low, recognize that it is greater than no confidence and collaboratively work toward new strategies to reach higher confidence. This might require a slight change in the plan or an entirely different action item. This is also an opportunity to problem‐solve barriers to change (eg, lack of time) and devise strategies (eg, starting with small changes) for responding to resistance.^15^ It may be useful to offer a behavioral menu if the patient is unsure about ideas to improve their confidence. Arrange accountability by encouraging the patient to set a specific time or date to report their progress.

Practitioner: On a scale of 0‐10, how sure are you that you will be able to accomplish your plan? I would like to make sure we've created a realistic and achievable goal.

Patient: 8

Practitioner: Great! How will you check in or follow up with your progress?

Patient: At my next appointment (2 weeks).

Step 4. Follow‐up

This step involves a review of the plan, reassurance, and next steps.^9^ If the patient reports completion or partial completion of their plan, clinicians should recognize their accomplishment. If the patient does not carry out their plan, we should provide reassurance that this is a common occurrence and ask, “What would you like to do next?”. The next step might be a modification of the existing plan or a new plan altogether.^9^ A recent guideline for persistent low back pain suggests patient reassessment at every visit using the self‐rated recovery question. This question asks, “How well do you feel you are recovering from your injuries?” The response options include (a) completely better, (b) much improved, (c) slightly improved, (d) no change, (e) slightly worse, (f) much worse, and (g) worse than ever. Patients reporting to be “completely better” or “much improved” should be considered recovered. Patients who have not recovered are advised to follow the care pathway outlined in this guideline.^3^

### 2.3 Treatment outcomes

After completing a cycle of brief action planning spanning four sessions over two months, the patient reported much improvement in low back symptoms and fewer recurrences of pain. She obtained full‐time employment during the course of treatment, working full duties with fewer missed days due to back pain. After initially returning to work for three consecutive full days, she reported feeling “more resilient” and identified useful self‐management strategies such as taking breaks (pacing) and stretching. Upon follow‐up, she reported a numeric pain rating score of 2/10.

## 3 DISCUSSION

The purpose of this case report is to illustrate how brief action planning was used for a patient with persistent nonspecific LBP as a structured and concise approach to help manage persistent psychosocial barriers. Psychosocial barriers or “Yellow Flags” can delay or impede recovery from musculoskeletal conditions, including LBP. Therefore, it is recommended that poor prognostic factors are identified early in the patient encounter.^3^ Healthcare providers can benefit from learning and implementing approaches to help patients with self‐management strategies and brief action planning is one potential strategy, which helped this patient improve self‐efficacy and physical activity levels.

Studies on brief action planning suggest that it offers benefits to some patients with low physical activity levels or low treatment adherence. In a previous study, patients with low levels of physical activity were more likely to achieve significant improvements in levels of physical activity following brief action planning.^9^ It is also possible that brief action planning contributes to treatment adherence as part of a multifactorial intervention.^16^ In addition, individuals with knee pain receiving brief action planning report that they complete preventive exercises more frequently.^17^ In our case study, brief action planning was able to improve self‐efficacy in this patient with LBP experiencing psychosocial barriers, warranting further research in its role for the management of LBP.

### 3.1 Limitations and future research

There are limitations to this case report. First, valid and reliable patient‐reported outcome measures were not used to assess changes in disability or quality of life over time. However, a numeric rating scale was used to measure improvements in pain. Second, this single case report does not provide findings on effectiveness; however, it does illustrate the clinical application of brief action planning for a patient with persistent LBP. There is moderate evidence that self‐management strategies have small effects on people with LBP.^18^ However, we were unable to locate any studies evaluating the effects of brief action planning on people with LBP. Future studies exploring the effects of brief action planning for patients with LBP who are experiencing psychosocial barriers are warranted. Future studies may assess whether brief action planning is an effective goal setting strategy in patients with persistent LBP, as well as assess which patients are best suited to participate in action planning.

Healthcare providers may consider the integration of evidence‐based interventions in their program of care for the management of patients presenting with psychosocial barriers. The clinician (chiropractor with 6 years in practice) in this study learned brief action planning from short online instructional videos and reading recent literature on the topic.^9^, ^14^, ^15^ Formal training can be obtained through the Centre for Collaboration, Motivation & Innovation.^19^ Formal training is not required to perform brief action planning, but if healthcare providers are uncomfortable or inexperienced implementing interventions to address psychosocial barriers, they may consider referring individuals at risk for poor long‐term LBP recovery to an appropriate provider.^16^ When patients present with poor prognostic factors that may delay recovery, self‐management interventions should be included into a plan of management.^3^ Emphasizing goal setting and active coping strategies may minimize reliance on passive therapies in persistent LBP. The key message in this case report is to evaluate and address poor prognostic factors early in the care plan in patients experiencing persistent nonspecific LBP. We have demonstrated how to do this using a specific method—brief action planning.

## CONFLICT OF INTEREST

None to declare.

## AUTHOR CONTRIBUTIONS

GC: involved in management of the case, collected clinical information, conceived the project, wrote the first draft of the manuscript, and approved the final draft. LV: conceived the project, edited the manuscript, and approved the final draft. CC: conceived the project, edited the manuscript, and approved the final draft. JJW, HY, and HMS: edited the manuscript and approved the final draft.

## CONSENT FOR PUBLICATION

Written informed consent was obtained from the patient for publication of this case report.

## References

[1] Cassidy JD , Carroll LJ , Côté P . The Saskatchewan health and back pain survey. The prevalence of low back pain and related disability in Saskatchewan adults. Spine. 1998;23(17):1860‐1866. 9762743 10.1097/00007632-199809010-00012

[2] Bath B , Trask C , McCrosky J , Lawson J . Demographic and health characteristics of rural‐ and urban‐dwelling canadians with chronic back disorders A population‐based comparison. Spine. 2014;39(23):1960‐1968. 25365711 10.1097/BRS.0000000000000561

[3] Wong JJ , Côté P , Sutton DA , et al. Clinical practice guidelines for the noninvasive management of low back pain: a systematic review by the Ontario Protocol for Traffic Injury Management (OPTIMa) Collaboration. Eur J Pain. 2016;21(2):201‐216. 27712027 10.1002/ejp.931

[4] Dunn KM , Jordan KP , Croft PR . Contributions of prognostic factors for poor outcome in primary care low back pain patients. Eur J Pain. 2010;15:313‐319. 20728385 10.1016/j.ejpain.2010.07.008 PMC3062783

[5] Eilayyan O , Thomas A , Hallé M‐C , et al. Promoting the use of self‐management in novice chiropractors treating individuals with spine pain: The design of a theory‐based knowledge translation intervention. BMC Musculoskelet Disord. 2018;19(1):328‐413. 30205825 10.1186/s12891-018-2241-1 PMC6134709

[6] Main CJ , George SZ . Psychologically informed practice for management of low back pain: future directions in practice and research. Phys Ther. 2011;91:820‐824. 21451091 10.2522/ptj.20110060

[7] Qaseem A , Wilt TJ , McLean RM , et al. Noninvasive treatments for acute, subacute, and chronic low back pain: a clinical practice guideline from the American college of physicians. Ann Intern Med. 2017;166(7):514‐U142. 28192789 10.7326/M16-2367

[8] Bussières AE , Al Zoubi F , Quon JA , et al. Fast tracking the design of theory‐based KT interventions through a consensus process. Implement Sci. 2015;10(1):18. 25880218 10.1186/s13012-015-0213-5 PMC4330935

[9] Gutnick D , Reims K , Davis C , et al. Brief action planning to facilitate behaviour change and support patient self‐management. J Clin Outcome Manag. 2014;21(1):17‐29.

[10] van Tulder MW , Assendelft WJJ , Koes BW , Bouter LM . Spinal radiographic findings and nonspecific low back pain ‐ a systematic review of observational studies. Spine. 1997;22:427‐434. 9055372 10.1097/00007632-199702150-00015

[11] O'Sullivan PB , Caneiro JP , O'Sullivan K , et al. Back to basics: 10 facts every person should know about back pain. Br J Sports Med. 2020;54(12):698‐699. 31892534 10.1136/bjsports-2019-101611

[12] Koka A , Hagger MS . A brief intervention to increase physical activity behavior among adolescents using mental simulations and action planning. Psychol Health Med. 2017;22(6):701‐710. 27427434 10.1080/13548506.2016.1211298

[13] Bussières AE , Stewart G , Al‐Zoubi F , et al. Spinal manipulative therapy and other conservative treatments for low back pain: a guideline from the Canadian chiropractic guideline initiative. J Manipulative Physiol Ther. 2018;41:265‐293. 29606335 10.1016/j.jmpt.2017.12.004

[14] Stott N , Rollnick S , Rees M , Pill R . Innovation in clinical method: diabetes care and negotiating skills. Fam Pract. 1995;12:413‐418. 8826057 10.1093/fampra/12.4.413

[15] Bean MK , Biskobing D , Francis GL , Wickham E . Motivational interviewing in health care: results of a brief training in endocrinology. J Grad Med Educ. 2012;4(3):357‐361. 23997882 10.4300/JGME-D-11-00166.1 PMC3444191

[16] Farmer AJ , Oke J , Hardeman W , et al. The effect of a brief action planning intervention on adherence to double‐blind study medication, compared to a standard trial protocol, in the Atorvastatin in Factorial with Omega EE90 Risk Reduction in Diabetes (AFORRD) clinical trial: a cluster randomised sub‐study. Diabetes Res Clin Pract. 2016;120:56‐64. 27522034 10.1016/j.diabres.2016.07.004 PMC6078174

[17] Koh LH , Hagger MS , Goh VHH , Hart WG , Gucciardi DF . Effects of a brief action and coping planning intervention on completion of preventive exercises prescribed by a physiotherapist among people with knee pain. J Sci Med Sport. 2017;20(8):723‐728. 28396206 10.1016/j.jsams.2017.02.008

[18] Oliveira VC , Ferreira PH , Maher CG , Pinto RZ , Refshauge KM , Ferreira ML . Effectiveness of self‐management of low back pain: systematic review with meta‐analysis. Arthritis Care Res (Hoboken). 2012;64:1739‐1748. 22623349 10.1002/acr.21737

[19] Brief Action Planning – CCMI [Internet] . Centrecmi.ca. 2020 https://centrecmi.ca/brief‐action‐planning/ Accessed 6 July, 2020

